# Delayed Diagnoses of *SGCE* Myoclonus-Dystonia

**DOI:** 10.5334/tohm.334

**Published:** 2020-07-28

**Authors:** M. Georgeta Varga, Nikita P. Nand, Mark S. LeDoux

**Affiliations:** 1 Parkinson Disease Movement Disorders Clinic, Austin, Texas, US; 2 The University of Texas Health Science Center at Houston, Houston, Texas, US; 3 University of Memphis, and Veracity Neuroscience LLC, Memphis, Tennessee, US

**Keywords:** *SGCE*, myoclonus, dystonia, tics, cerebral palsy, Tourette syndrome

## Abstract

**Background:**

Myoclonus-dystonia due to *SGCE* mutations (OMIM: 159900) most commonly presents during childhood with mainly upper body myoclonus, and mild dystonia affecting the neck and arms.

**Case reports:**

Herein, we report patients misdiagnosed during childhood with Tourette syndrome and dyskinetic cerebral palsy, and, during adulthood, found to harbor *SGCE* frameshift mutations.

**Discussion:**

Myoclonus-dystonia may be underdiagnosed due to phenotypic misclassification during childhood. *SGCE* mutations should be included in the differential diagnosis of childhood movement disorders that ostensibly manifest with tics, myoclonus, or abnormal posturing secondary to dystonia and/or spasticity.

**Highlights:**

Due to pleiotropy, variable penetrance, broad differential, and hereditary effects of imprinting, the diagnosis of a disorder of childhood onset, myoclonus-dystonia due to *SGCE* mutations, may be delayed until adulthood, often compromising appropriate clinical management and genetic counseling.

Mutations in *SGCE* (Chr 7q21.3), a maternally imprinted gene, have been causally linked to Myoclonus-Dystonia syndrome (M-D, DYT-*SGCE*, OMIM: 159900) [1]. Classically and most commonly, this disorder is characterized by childhood onset of myoclonus and dystonia [2]. Patients typically present with upper-body predominant, alcohol-responsive myoclonic jerks, along with cervical and arm dystonia [2]. Psychiatric co-morbidities including depression, anxiety, bipolar disorder, phobias, alcoholism, and obsessive-compulsive disorder have also been associated with M-D and tend to worsen over the disease course [3,4]. These psychiatric manifestations may occur in isolation in some members of affected pedigrees. In addition, the neuropsychiatric spectrum of *SGCE* mutations is broad and may include early gait dysfunction with leg involvement [5] and, more rarely, cognitive dysfunction [6]. Penetrance is incomplete and notable intrafamilial and extrafamilial phenotypic variability is well established [7]. Early and correct diagnosis may facilitate appropriate pharmacological and surgical interventions, and genetic counseling. We present two cases of genetically confirmed *SGCE* myoclonus dystonia, initially misdiagnosed as Tourette syndrome (Case 1) and dyskinetic cerebral palsy (Case 2), respectively.

Case 1. A 32-year-old right-handed female presented to our movement disorders clinic with abnormal movements with onset at 7 years of age. Historically, these movements were described as involuntary, brief, jerky movements of the hands, shoulders, neck and trunk. These movements were previously classified as tics by prior treating neurologists, and she had been diagnosed with Tourette syndrome. During her adult years, she recognized that her involuntary movements were exacerbated by caffeine intake and improved with alcohol. She reported a history of abnormal vocalization described as “throat clearing” but compatible with laryngeal myoclonus. There was no history of head trauma or neuroleptic use. Neuropsychiatric review of systems was pertinent for mild anxiety of 20 years’ duration. Family history was significant for reported “tic-like” movements in her father and obsessive-compulsive disorder (OCD) in her mother (Figure 1, Pedigree 1). Previously, she had been treated with tetrabenazine 12.5 mg PO TID and clonazepam 0.5 mg PO BID. Tetrabenazine was not tolerated due to worsened anxiety. Clonazepam showed no significant clinical benefit. On neurological examination, action myoclonus of the arms and trunk was apparent. There was no dystonia, motor tics, phonic tics, or phonic myoclonus. There has been a greater than 50% improvement in myoclonus with deutetrabenazine (6 mg PO BID) The positive response to deutetrabenazine has been sustained for over one year and the patient has not been interested in taking higher dosages of deutetrabenazine. Testing for an *SGCE* mutation was pursued given the characteristic historical and visual presence of upper body action myoclonus of childhood onset, positive family history of a movement disorder and OCD, and alcohol responsiveness. Bidirectional Sanger sequencing identified a heterozygous single base pair deletion (GRCh38/hg38, NC_000007.14: g.94603404delT, NM_001099401.1(*SGCE*_v001): c.711delA, p.Glu238Lysfs*9). This frameshift mutation is predicted to alter the length of the ε-sarcoglycan reference protein of 462 residues to a protein of 245 residues. The location of the mutation most likely leads to nonsense mediated decay of *SGCE* transcripts.

**Figure 1:**
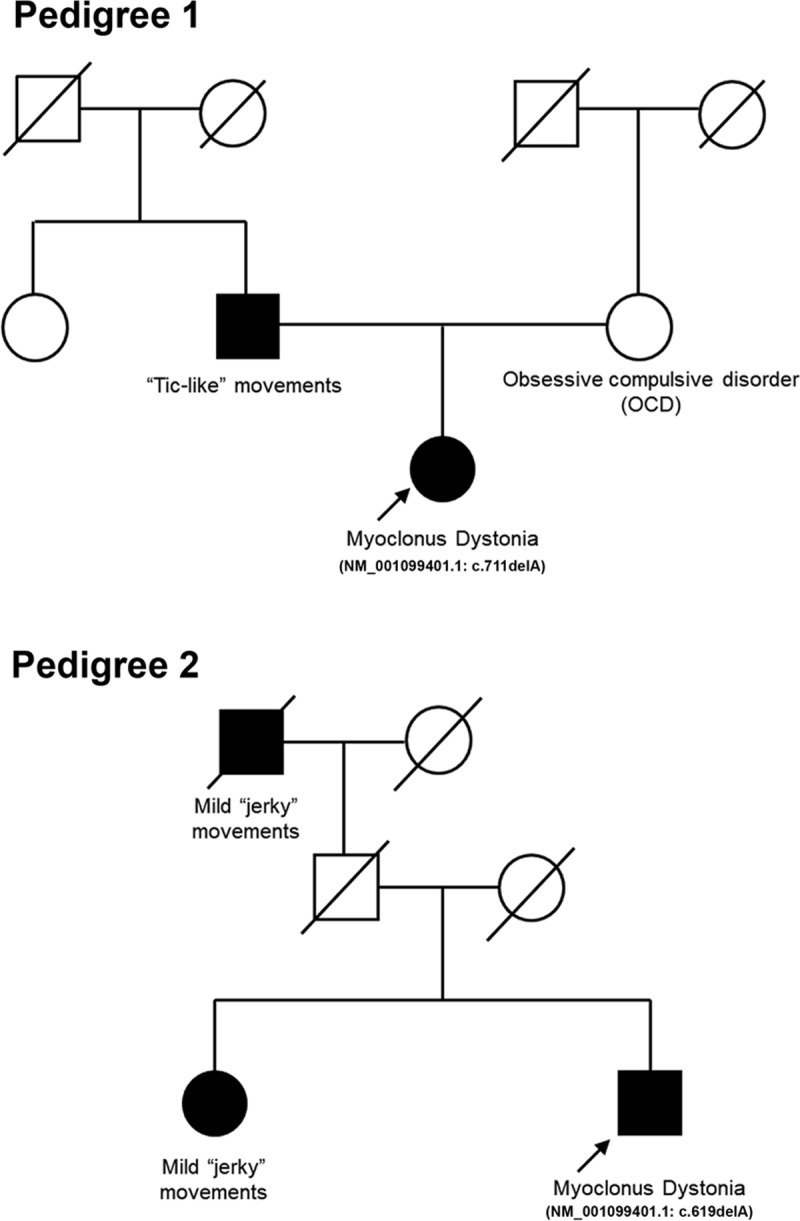
**Pedigree 1:** I, II, III Generation, Full symbols symptomatic individuals, Slashed symbols deceased individuals, II-2 Father with tic-like movements, III-3 Mother with OCD, III-1 Patient with + DyT11. **Pedigree 2:** I, II, III Generation, Full symbols symptomatic individuals, Slashed symbols deceased individuals, I-1 Paternal Granfather with tic-like movements, deceased, III-1 Sister with jerky movements, III-2 Patient with + DyT11.

Case 2. A 64-year-old left-handed male presented to our movement disorders clinic with a history of involuntary movements since childhood and mild developmental delay. He was delivered by Cesarean section (C-section) at 7 months of gestation due to fetal distress and was diagnosed with pulmonary hypoplasia. However, he experienced an unremarkable early postnatal course. He had mildly delayed gross and fine motor milestones without intellectual disability. At 8 years of age, he began to manifest involuntary “jerky” movements mainly involving his upper extremities. During childhood and early adulthood, he was assessed by two independent neurologists. Diagnostic work-up included a normal brain magnetic resonance imaging (MRI) brain scan and normal electroencephalogram. One neurologist provided a provisional diagnosis of dyskinetic cerebral palsy. Reportedly, the “jerky” movements were mild but persistent over the subsequent four decades and did not interfere with physical activity. However, at age 45, he experienced worsening of clinical signs with more pronounced “jerking” of his arms, neck and shoulders. Changes in speech with a weak and breathy voice that improved with whispering were also reported. Amelioration of symptoms could only be achieved by drinking alcohol. He had long-standing mild generalized anxiety. Previous treatment with phenobarbital, levetiracetam, and clonazepam had been ineffective. Family history was significant for mild “jerky” movements in his sister and paternal grandfather (Figure 1, Pedigree 2). Clinical examination was notable for spontaneous myoclonic movements involving the axial and shoulder girdle muscles and arms that worsened with activity. Additionally, mild cervical dystonia and mixed vocal dystonia (clinically pharyngeal and laryngeal involvement) was noted. However, this patient was not assessed with laryngoscopy and videostroboscopy. The remainder of the general physical and neurological examinations was unremarkable. Deutetrabenazine caused fatigue and was of no significant clinical benefit at a dosage of 9 mg PO BID. Given his characteristic myoclonic and dystonic features on clinical examination, positive family history, and response to alcohol, *SGCE* was assessed with bidirectional Sanger sequencing which permitted identification of a heterozygous single base pair deletion (GRCh38/hg38, NC_000007.14: g.94618801delT, NM_001099401.1(*SGCE*_v001): c.619delA, p.Arg207Glyfs*12). Rather than translation of a truncated protein of 217 residues, the location of the stop codon most likely precipitates nonsense mediated decay of *SGCE* transcripts. This patient has been offered deep brain stimulation (bilateral globus pallidus internus) for treatment of his myoclonus-dystonia.

## Discussion

Unfortunately, our two cases illustrate the poignant realty that the diagnosis of childhood onset myoclonus-dystonia can be delayed for decades. The delayed diagnoses in our two pedigrees also suggests that myoclonus-dystonia and *SGCE*-associated neuropsychiatric disorders may be significantly underdiagnosed. For patients with a characteristic clinical picture, *SGCE* mutations are the most common known etiology for this neuropsychiatric disorder [8] and genetic testing should be pursued in these cases unless limited by financial considerations. *SGCE*-linked myoclonus-dystonia may show clinical similarities to tics, spasticity, dystonic tremor, epilepsy, and conversion disorders with positive motor features. While characterized by mainly upper body (arms and trunk) myoclonus and mild focal or segmental dystonia (neck, distal arms), a broad array of atypical neuropsychiatric presentations and manifestations are well documented in the existing literature [9].

Use of a published diagnostic algorithm for myoclonus [10], and then diagnostic criteria for M-D [8], are the first steps in differentiating M-D from other neurological disorders. Generally, in patients with M-D, myoclonus predominates over dystonia and onset is prior to adulthood. Our two patients had been incorrectly diagnosed with Tourette syndrome and dyskinetic cerebral palsy. In contrast to myoclonus, tics are typically more complex, suppressible for brief periods, and often precipitated by a premonitory urge. Brain MRI is frequently abnormal in dyskinetic cerebral palsy and the dystonic and choreoathetoid movements characteristic of this disorder are usually much slower than myoclonus. The developmental time course of disease manifestations can also help to distinguish M-D from other pediatric movement disorders. In this regard, some M-D patients exhibit mild lower extremity dystonia and associated gait dysfunction during childhood or adolescence that subsequently resolves [11].

There are other potential reasons for a delay in diagnosis. Maternal imprinting and intrafamilial phenotypic variability can complicate interpretation of family history and lead to dismissal of hereditary etiologies in differential diagnoses. Although useful historical information, the response to consumption of alcoholic beverages is uncommonly available in children and adolescents. In some cases, no connection is made between the psychiatric and motor phenotypes, which may be considered as separate diseases. Neurological manifestations, particularly dystonia, may be subtle and mild psychiatric manifestations such as anxiety and depression may have not undergone treatment for decades. Finally, dystonia can be present in childhood and then abate.

Delayed diagnosis of M-D can have a significant impact on patients and their families. In particular, a prompt etiological diagnosis can inform treatment decisions and caregiver concerns [12]. M-D may benefit from occupational and physical therapy, and often responds to specific pharmacological interventions (tetrabenazine, anticholinergics, and zonisamide) and electrical stimulation of the globus pallidus [8,13,14,15], and, as reported here, deutetrabenazine may be an additional therapeutic option. Earlier surgical intervention in patients with severe disease and inadequately responsive to oral pharmacotherapy and/or injections of botulinum toxin may improve long-term outcomes [16]. Earlier diagnosis may reduce alcoholism [4] and prompt recognition of psychiatric co-morbidities allowing these to be symptomatically managed before they exert deleterious effects on academic performance, work productivity and social interactions. Prompt etiological diagnosis of M-D may also facilitate academic, career and family planning [12].

Genetic testing should begin with Sanger or next-generation sequencing and, ideally, confirmation with bidirectional Sanger sequencing. Sequencing should cover all coding exons, exon-intron boundaries, and the core promoter region of *SGCE*. Genetic testing should also include exclusion of exonic, multi-exonic and interstitial deletions, particularly in patients with typical clinical presentations and absence of single nucleotide changes or small indels [17]. Genetic testing for *SGCE* mutations should be considered in “scan normal” cases of putative cerebral palsy [18]. Genetic counseling should include consideration of maternal imprinting, pleiotropy, and variable penetrance.
